# Single Center Experience on Endovascular Treatment of Occlusion and/or Stenosis of Bilateral Carotid Arteries

**DOI:** 10.1002/brb3.70395

**Published:** 2025-03-26

**Authors:** Şule Dalkiliç, Sena Boncuk Ulaş, Levent Avci, Beyza Nur Bozkurt, Alihan Abdullah Akbaş, Vasfiye Sezer, Esra Ünal, Ayşe Kristina Polat, Semanur Aksu, Derya Kara Genç, Halil Alper Eryilmaz, Türkan Acar, Yeşim Guzey Aras, Bilgehan Atilgan Acar

**Affiliations:** ^1^ Department of Neurology Sakarya Training and Research Hospital Sakarya Turkey; ^2^ Independent Researcher Dunkerque France; ^3^ Department of Neurology Sakarya University Faculty of Medicine Sakarya Turkey

**Keywords:** bilateral carotid artery stenosis, carotid artery occlusion, stroke

## Abstract

**Introduction:**

Carotid artery stenosis is the presence of 50% or more stenosis in the internal carotid artery (ICA) according to the North American Symptomatic Carotid Endarterectomy Trial (NASCET) criteria and is one of the leading etiological factors of ischemic stroke. The severity of stenosis is associated with stroke risk. The prevalence of bilateral carotid artery stenosis (BCAS) varies. This study aims to evaluate the clinical outcomes and complications associated with carotid artery stenting in patients with BCAS or occlusion in a comprehensive stroke center.

**Methods:**

The data of patients who underwent carotid artery stenting (CAS) between January 2020 and September 2024 were scanned. The demographic data and comorbidities were noted from the patients’ files. Then, the patients were divided into two groups. The first group is “bilateral 50%–99% ICA stenosis” and the second group is “one side ICA occluded contralateral side ICA ≥50% stenosis”. Both groups were also listed as symptomatic and asymptomatic. Demographic and endovascular procedure data were analyzed.

**Results:**

A total of 82 patients were included in this study, 69 male (84.14%) and 13 female (15.85%). The mean age was 69.46 ± 7.24. Hypertension (HT) was the most common comorbid disease (69.51%). Sixty‐six patients (80.50%) were symptomatic and 16 (19.50%) were asymptomatic. TIA developed in one patient in the first group. Two minor (The National Institutes of Health Stroke Scale [NIHSS] 1–3) and two major strokes developed in the second group. Only three patients (two in the first group, one in the second group) underwent postdilatation balloon angioplasty.

**Conclusion:**

Endovascular treatment seems to be an acceptable strategy in comprehensive stroke centers where the possible complication risks can be well managed in this group of patients.

## Graphical abstract

This study investigates the role of carotid artery stenting (CAS) in the treatment of bilateral carotid artery stenosis and/or occlusion. Although transient ischemic attacks (1.22%) and minor strokes (2.44%) were observed, major strokes (2.44%) were effectively treated with mechanical thrombectomy. These results demonstrate that endovascular treatment is a safe and effective strategy for the treatment of bilateral carotid lesions in high‐risk patients, with complications well managed in a comprehensive stroke center setting. However, future randomized trials are needed to define optimal treatment protocols and improve patient outcomes.

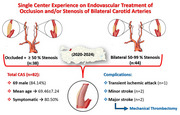

## 1 Introduction

Carotid artery stenosis is defined as the presence of 50% or more stenosis in the extracranial internal carotid artery (ICA) according to the North American Symptomatic Carotid Endarterectomy Trial (NASCET) (Ferguson et al. 1999) criteria and is one of the leading and important etiological factors of ischemic stroke (Naylor et al. 2023; Oshita et al. 2020). According to NASCET data, 50%–69% ICA stenosis is classified as moderate and 70%–99% ICA stenosis as severe (Ferguson et al. 1999). The severity of ICA stenosis is associated with stroke risk, and hemodynamically significant ICA stenosis is responsible for 12%–20% of all ischemic strokes (Oshita et al. 2020; Leszczyński et al. 2020). The prevalence of ICA stenosis has been reported to be approximately 1.5% for 30–79 ages in studies, and the prevalence increases especially with advanced age and may reach up to 12% (Diao et al. 2016; Khan et al. 2021). The prevalence of bilateral carotid artery stenosis (BCAS) varies in the literature, but one study emphasized that BCAS was detected in 10% of patients who underwent carotid ultrasonography (USG) screening before coronary artery bypass surgery (Song et al. 2020). The annual stroke risk in patients with BCAS ranges between 0% and 13% (Wanamaker et al. 2012; Rijbroek et al. 2006).

According to the 2023 ESVS/ESC Guideline, lifestyle modification, carotid endarterectomy (CEA), and carotid angioplasty‐stenting (CAS) are recommended as treatments for ICA stenosis. In symptomatic patients, lifestyle modification and CEA are recommended as Class IA for patients with 70%–99% stenosis and CAS as Class IIa Level B in patients at high risk for CEA. In symptomatic patients with 50%–69% stenosis, lifestyle change and CEA are recommended as Class IIa Level B (Naylor et al. 2023). Studies have reported that CAS and CEA are performed in approximately one hundred thousand patients annually in Europe and North America, half of whom are symptomatic and half asymptomatic (Amin 2015). Both interventional procedures have advantages and disadvantages, and the appropriate selection of patients in the perioperative period is of great importance. In studies, fatal and disabling event rates of both CAS and CEA are reported to be equal, while nondisabling procedural stroke rates are slightly higher for CAS (Amin 2015). The most common complications associated with CEA performed under general anesthesia include anesthesia and surgery‐related complications, as well as recurrent laryngeal nerve palsy (Halliday et al. 2021; Dorobisz et al. 2024).

Bilateral ICA stenosis/occlusion is rare but of great importance in terms of the stroke risk they carry. When the studies conducted in this field in the literature are examined, it is seen that there are no randomized controlled studies and limited articles in which a small number of patient series are presented (Oshita et al. 2020; Rijbroek et al. 2006; Kocayiğit et al. 2021; Veselka et al. 2011). Uncertainties regarding the follow‐up and treatment of these patients create a need for new studies in this field.

Here, we aimed to evaluate the demographic data, risk factors, treatment processes, and complications of patients with angiographically detected bilateral ICA stenosis/occlusion who underwent CAS in a comprehensive stroke center and to discuss the results in the light of the literature.

## 2 Material and Methods

The data of patients who underwent CAS at the Neurology Clinic of Sakarya University Faculty of Medicine Education and Research Hospital between January 2020 and September 2024 were retrospectively scanned for the study. Before starting the study, approval was obtained from the ethics committee of our university on November 29, 2023 with the approval number E‐71522473‐050.01.04‐309427‐340. Patients who were detected to have at least 50% bilateral ICA stenosis and/or unilateral ICA occluded and at least 50% stenosis on the contralateral side ICA on digital subtraction angiography (DSA) and underwent CAS were included in the study. After obtaining diagnostic images on DSA, patient selection for CAS versus CEA was based on a comprehensive, interdisciplinary evaluation and decided in a council with cardiovascular surgery. Factors such as lesion characteristics (e.g., degree of stenosis, plaque morphology, angulation, and calcification), aortic arch anatomy, contralateral carotid lesions, comorbid conditions, and patient preferences were considered to determine the optimal treatment approach (Çiçek et al. 2024). When making the CAS decision, the criteria of ≥50% stenosis according to NASCET data for symptomatic patients, and stenosis progression of more than 20% and/or impaired cerebral vascular reserve and/or detection of ipsilateral silent infarcts for asymptomatic patients were considered. Patients considered suitable for CEA were referred to surgery. Patients who underwent only unilateral CEA or bilateral CEA were not included in the study. The exclusion criteria of the study were patients with anatomical conditions unsuitable for the procedure, those who declined to undergo the procedure, asymptomatic patients with renal failure not requiring dialysis, and patients who were unable to attend outpatient clinic follow‐ups. Of the 85 patients who met the criteria, 2 were excluded because they did not accept any procedure, 1 was excluded because their outpatient clinic follow‐up notes could not be accessed, and 82 patients were included in the study.

The patients’ age, gender, comorbidities, presence of atrial fibrillation (AF), hyperlipidemia (HPL), and antiaggregant and anticoagulant medication information were noted from the patients’ files. According to the 2023 ESVS/ESC Guidelines, LDL > 70 mg/dL was accepted as the criterion for HPL diagnosis (Naylor et al. 2023). Then, the patients were divided into two groups. The first group included those with “bilateral 50%–99% ICA stenosis”, and the second group included those with “one side ICA occluded contralateral side ICA ≥50% stenosis”. Both groups were also listed as symptomatic and asymptomatic. In the first group, patients with a history of anterior cerebral circulation‐related stroke or transient ischemic attack (TIA) within the last 6 months were considered symptomatic on the side of carotid stenosis (Naylor et al. 2023). In the second group, since one side of the ICA was occluded, having had any stroke or TIA from the right or left hemisphere related to anterior cerebral circulation within the last 6 months was considered symptomatic. Routinely performed brain computed tomography (CCT), cervical CT‐angiography (CCTA), and carotid‐vertebral artery Doppler USG results were obtained from the files of all patients before the procedure, but the data measured during the endovascular procedure were used for exact stenosis rates.

All endovascular procedures were performed by an interventional neurologist. Before the procedure, all patients received acetylsalicylic acid (300 mg), clopidogrel (600 mg), and atorvastatin (40 mg) in a loading dose. In the CAS procedure, all patients underwent femoral access. All patients received unfractionated heparin (100 IU/kg body weight). The procedure was initiated with a diagnostic angiogram. After confirming the severity and morphology of the stenosis, the common carotid artery (CCA) was accessed with a 90 cm 6F sheath. After the distal filter was opened, predilatation balloon angioplasty was performed with a suitable‐sized balloon. In patients in whom the distal filter protection was not used due to anatomic reasons, the procedure was continued with a soft wire without a filter. Atropine (≤1.0 mg) was administered intravenously to prevent severe bradycardia during dilatation. Then, a suitable‐sized Carotid Wallstent was placed between the ICA and CCA to cover the lesion. The distal filter was then removed and the angiogram was completed including the intracranial arteries. After the procedure, images were taken again, and stent patency, presence of residual stenosis, and distal embolism were recorded. Postdilatation balloon angioplasty was performed in patients with more than 30% residual stenosis. Dual antiplatelet therapy (clopidogrel [75 mg/day] and acetylsalicylic acid [100 mg/day]) was applied for at least 3 months after the procedure. Atorvastatin (20–40 mg/day) was also added to the treatment in those with HPL. Sheath site‐related complications that developed after the procedure, TIA, minor stroke, major stroke, carotid artery dissection, hyperperfusion syndrome, renal dysfunction, hemodialysis requirement, and acute coronary syndrome events were obtained from the 3rd and 6th month outpatient clinic follow‐up notes.

Statistical evaluation was performed using the Statistical Package for Social Sciences (SPSS) 22.0 package program. Mean, standard deviation, median, lowest and highest value, frequency, and ratio values were used in the descriptive statistics of the data.

## 3 Results

A total of 82 patients were included in the study, 69 male (84.14%) and 13 female (15.85%). The mean age of all patients was 69.46 ± 7.24. When comorbid diseases were examined, hypertension (HT) was the most common with 69.51%, while 68.29% of patients had stroke, 36.58% had diabetes mellitus (DM), 35.36% had coronary artery disease (CAD), 6.09% had peripheral artery disease (PAD), and 4.87% had acute or chronic renal failure (AKI/CKD). AF was detected in 3.65% of patients. HPL was seen with a frequency of 62.19%, while the rate of patients with a history of smoking was 24.39%. Antiplatelet and anticoagulant medication use was detected as 67.07% and 4.87%, respectively. Of the 82 patients, 66 (80.50%) were symptomatic and 16 (19.50%) were asymptomatic (Table 1). There were a total of 38 patients with occluded ICA on one side and ≥50% stenosis on the contralateral ICA, and all of them underwent CAS. Thirty‐two of these patients were symptomatic and 6 were asymptomatic, but all of them had more than 20% progression in stenosis rates in outpatient follow‐up CT‐A and/or Doppler USG performed within 6 months. In the bilateral 50%–99% ICA stenosis group, there were 44 patients, 34 of whom were symptomatic, and 10 of whom were asymptomatic. Unilateral CAS was performed in 14 of these 44 patients. It was determined that 28 patients underwent bilateral CAS between 2020 and 2024 (Table 2). The closest hospitalization period for the second CAS procedure was noted as 1 month and the most distant as 38 months. In this group, 2 of the 44 patients underwent CAS to one ICA and CEA to the contralateral ICA. It was learned from the files of the patients in the bilateral 50%–99% ICA stenosis group who underwent bilateral procedures (CAS and/or CEA) that cardioembolism exclusion (echocardiography [ECG], 72‐h rhythm Holter) was performed during both hospitalizations.

**TABLE 1:** Demographic and clinical characteristics of patients.

Age, mean ± SD	69.46 ± 7242
Male sex, *n* (%)	69 (84.14%)
Stroke, *n* (%)	56 (68.29%)
Coronary artery disease, *n* (%)	29 (35.36%)
Hypertension, *n* (%)	57 (69.51%)
Diabetes mellitus, *n* (%)	30 (36.58%)
Peripheral artery disease, *n* (%)	5 (6.09%)
Atrial fibrilation, *n* (%)	3 (3.65%)
ARF/CRF, *n* (%)	4 (4.87%)
Hyperlipidemia, *n* (%)	51 (62.19%)
Smoking, *n* (%)	20 (24.39%)
Antiaggregant medication use, *n* (%)	55 (67.07%)
Anticoagulant medication use, *n* (%)	4 (4.87%)
Symptomatic, *n* (%)	66 (80.50%)

Abbreviations: ARF, acute renal failure; CRF, chronic renal failure; SD, standard deviation.

**TABLE 2:** Endovascular procedure data.

		Occluded + ≥%50 stenosis (*n*:38)		Bilateral %50–99 stenosis (*n*:44)		Total (*n*:82)
		Symptomatic (*n*:32)	Asymptomatic (*n*:6)	Symptomatic (*n*:34)	Asymptomatic (*n*:10)	
Procedure type^a^	CAS	32	6	11	3	52
	CAS + CAS	—	—	22	6	28
	CAS + CEA	—	—	1	1	2
Complication^b^	Sheat site‐related complications	0	0	0	0	0
	TIA	1	0	0	0	1
	Minor stroke (NIHSS 1–3)	0	0	1	1	2
	Major stroke (NIHSS > 3)	0	0	2^c^	0	2
Symptom to CAS time	0–14 day	27	—	27	—	54
	15–30 day	5	—	7	—	12
Need for postdilation balloon angioplasty		2	—	1	—	3

Abbreviations: CAS, carotid artery stenting; CEA, carotid artery endarterectomy; NIHSS, The National Institutes of Health Stroke Scale; TIA, transient ischemic attack.

^a^ In our study, there were no patients who underwent only unilateral CEA or bilateral CEA.

^b^ Carotid artery dissection, hyperperfusion syndrome, renal dysfunction, hemodialysis requirement, and acute coronary syndrome were not observed during the endovascular procedure.

^c^ In two patients who underwent CAS on the symptomatic side, endovascular mechanical thrombectomy was performed due to postprocedure embolism and complete recanalization was achieved. One patient had mRS: 0, the other had mRS: 5.

When the complications were examined, TIA developed in one symptomatic patient in the first group. In the second group, two patients had minor strokes (The National Institutes of Health Stroke Scale [NIHSS] 1–3) (one symptomatic, one asymptomatic), and two symptomatic patients had major strokes (NIHSS > 3). In patients who developed major stroke, endovascular mechanical thrombectomy was applied immediately during the CAS procedure, and complete recanalization was achieved. However, one patient had complete recovery (mRS: 0) and the other had poor clinical outcome (3rd month mRS: 5). None of the patients had carotid artery dissection, hyperperfusion syndrome, renal dysfunction, hemodialysis requirement, or acute coronary syndrome during the endovascular procedure (Table 2).

A total of 54 symptomatic patients underwent the procedure within the first 14 days, and 12 symptomatic patients underwent the procedure within 14–30 days (Table 2).

All patients underwent CAS after predilatation balloon angioplasty, and only three patients with ≥30% residual stenosis underwent postdilatation balloon angioplasty (Table 2).

## 4 Discussion

ICA stenosis is one of the leading causes of ischemic stroke. In the literature, we see that the prevalence of atherosclerotic lesions in the carotid arterial system is approximately 27% over the age of 30. The risk of ICA stenosis and occlusion increases with age (Amin 2015). Although bilateral ICA stenosis/occlusion is a rare entity, it is of great importance because it increases the risk of ischemic stroke and poor outcomes (Wanamaker et al. 2012; Rijbroek et al. 2006; Lai et al. 2019). Although current guidelines recommend CEA with a high level of evidence in unilateral symptomatic carotid stenosis, they do not show CEA or CAS as superior to each other in patients with 50%–99% stenosis and contralateral carotid occlusion, and recommend it as Class IIa Level B (Naylor et al. 2023). CAS and CEA each offer distinct advantages and disadvantages, making patient‐specific decision‐making critical. CAS is less invasive, making it particularly advantageous for elderly patients with significant comorbidities or challenging anatomical considerations, such as a high carotid bifurcation or severe neck immobility. However, CAS is associated with a higher risk of perioperative stroke, especially in patients with tortuous vascular anatomy or significant aortic arch abnormalities. In contrast, CEA provides excellent long‐term durability and lower restenosis rates, but it involves a higher risk of cranial nerve injuries and myocardial infarction due to the surgical stress. Multidisciplinary collaboration is crucial to optimizing outcomes (Çiçek et al. 2024; Deniz et al. 2023; Vatan et al. 2023; Kirişçi 2020; Boncuk Ulaş and Acar 2025). Based on these recommendations, we created one of our groups from these patients. Our second group of cases consisted of patients with bilateral moderate‐severe (50%–99%) stenosis but without a clear treatment recommendation in the guidelines. For all these patients with bilateral ICA moderate‐severe stenosis and/or occlusion, the appropriate treatment should be selected individually considering the symptoms, plaque type, aortic arch, ICA anatomy, and comorbid diseases. Considering the complications of CEA in bilateral advanced ICA stenosis and patient preferences, CAS has become a reasonable option as a safe and effective procedure in bilateral carotid stenosis with the rapid development of the endovascular approach and the introduction of distal embolism prevention devices (Kocayiğit et al. 2021).

In a study conducted in 2020, the mean age of patients with BCAS who underwent endovascular procedures was reported as 70.3 ± 7.5 and 91% were male (Kocayiğit et al. 2021). Similarly, in our study, we found the mean age to be 69.46 ± 7.24 and 84.14% of the patients were male. HT, DM, HPL, and smoking are well‐known cardiovascular risk factors. In particular, the relationship between smoking and carotid artery disease and carotid stent restenosis has been shown in studies (Alagoz et al. 2016; Stoisavljevic et al. 2024). In our patients, the most common of these risk factors was HT with 69.51%, while HPL came second with 62.19%. Smoking history was present in 24.39% of the patients. In one study, bilateral and unilateral ICA stenoses were compared, and the history of stroke in these patients was found to be 40% in the group with bilateral stenosis and 38% in the group with unilateral stenosis (Oshita et al. 2020). In our study, the rate of previous stroke history was 68.29%, which may be due to the inclusion of patients with both bilateral and advanced stenosis. In another study conducted in 2023, severe CAD was detected in 64.7% of patients with bilateral ICA stenosis of 70% and above (Park et al. 2024). CAD was observed in 35.36% of the patients in our study. In the same study, HT was reported in 85.5%, HPL in 87.9%, and smoking history in 31.9%, and the results are similar to ours (Park et al. 2024).

When we look at the complications associated with CAS, a meta‐analysis of 10 retrospective studies reported the complications and their incidence rates after CAS in BCAS patients as follows; hyperperfusion syndrome 3.33%, stroke 3.20%, myocardial infarction 0.60%, and death 1.20% (Veselka et al. 2011). Another recent study evaluating the CAS procedure in 12 BCAS patients reported the rate of hyperperfusion syndrome as 0%, the ischemic lesion detection rate in magnetic resonance imaging (MRI) after the procedure as 37.5% in eight patients who underwent simultaneous bilateral CAS and 12.5% in four patients who underwent staged bilateral CAS, but stated that none of them had stroke symptoms (Oshita et al. 2020). CAS stands out as a less invasive and generally safer method, especially in a carefully selected patient group. However, despite the use of distal protection, ipsilateral ischemic lesions, which are observed at a higher rate compared to CEA, pose a problem for CAS. The characteristics of the plaque are also very important in this regard and a good preprocedural evaluation is required (Squizzato et al. 2023; Hokari et al. 2014). Our study, with a total of 82 patients, is the study with the largest BCAS data pool in the literature to our knowledge. Complications developed after the procedure in five of our patients, one of whom had TIA, two had minor strokes, and two had major strokes. Both major strokes developed due to embolism to the distal middle cerebral artery during the procedure and mechanical thrombectomy could be performed in these patients with immediate endovascular intervention. Because our center has the characteristics of a comprehensive stroke center. As a result, successful TICI 3 recanalization was achieved. Carotid artery dissection, hyperperfusion syndrome, hemodialysis, and myocardial infarction were not detected.

The ideal time recommended for interventional procedures for carotid artery stenosis in the 2023 ESVS/ESC guideline and studies in the literature is the first 14 days after symptoms (Naylor et al. 2023; Maleux et al. 2006). A study conducted in Sweden in 2015 divided patients who underwent CAS into four groups as 0–2, 3–7, 8–14, and 15 days and above and found no significant difference between the groups in terms of the risk of stroke and death within 30 days (Rothwell et al. 2004; Jonsson et al. 2015). In our study, we applied the CAS procedure to a total of 54 symptomatic patients within the first 14 days. The remaining 12 symptomatic patients underwent the procedure between 15 and 30 days. Of the five complications observed, one minor stroke and one major stroke were seen in the group that underwent the procedure between 15 and 30 days; one TIA, one minor, and one major stroke were seen in the group that underwent the procedure within the first 14 days.

Our study has several limitations that should be acknowledged to provide a balanced interpretation of the results. First, the retrospective design of the study may introduce biases related to data collection and interpretation. Additionally, the single‐center nature of the study limits the generalizability of the findings to other populations or settings. The potential for selection bias further complicates the robustness of the results. Moreover, the lack of long‐term follow‐up data restricts our ability to assess the sustainability of the outcomes over time. Finally, the absence of randomization in our study design limits the ability to draw definitive causal conclusions.

In conclusion, endovascular treatment seems to be an acceptable strategy in comprehensive stroke centers where the possible complication risks can be well managed in this group of patients, which operators avoid the procedure and which we do not encounter very often in the literature. It is obvious that successful results will be obtained with an appropriate treatment strategy for selected patients. Future randomized controlled trials are essential to better define the optimal treatment strategies for patients with BCAS/occlusion, particularly in high‐risk groups. Additionally, advancements in imaging techniques and patient‐specific risk stratification tools could significantly enhance the precision of treatment selection and improve outcomes in this complex patient population.

## Author Contributions

**Şule Dalkiliç**: conceptualization, data curation, formal analysis, visualization, writing–original draft. **Sena Boncuk Ulaş**: conceptualization, data curation, investigation, software, writing–original draft, writing–review, and editing. **Levent Avci**: conceptualization, data curation, formal analysis, visualization. **Beyza Nur Bozkurt**: formal analysis, software. **Alihan Abdullah Akbaş**: data curation, formal analysis. **Vasfiye Sezer**: investigation, methodology, validation. **Esra Ünal**: data curation, formal analysis. **Ayşe Kristina Polat**: investigation. **Semanur Aksu**: methodology, validation. **Derya Kara Genç**: project administration, software. **Halil Alper Eryilmaz**: data curation, investigation, software. **Türkan Acar**: methodology, investigation, project administration, software, validation, visualization. **Yeşim Guzey Aras**: investigation, methodology, validation. **Bilgehan Atilgan Acar**: conceptualization, data curation, investigation, methodology, project administration, software, supervision, writing–review, and editing.

## Disclosure

Due to the nature of the research and ethical reasons supporting data is not available.

## Ethics Statement

This study was approved by the Ethics Committee of Sakarya University Faculty of Medicine (approval no E‐71522473‐050.01.04‐309427‐340).

## Consent

Informed consent was obtained from all subjects involved in the study.

## Conflicts of Interest

The authors declare no conflicts of interest.

### Peer Review

The peer review history for this article is available at https://publons.com/publon/10.1002/brb3.70395.

## Data Availability

Data sharing is not applicable to this article.
